# Supplementary material for: Absolute Configuration and Biological Evaluation of Novel Triterpenes as Possible Anti-Inflammatory or Anti-Tumor Agents
Source: Molecules. 2022 Oct 6;27(19):6641. doi: 10.3390/molecules27196641 (PMC9572544; doi:10.3390/molecules27196641)
Supplement: Supplementary file 1 [file molecules-27-06641-s001.zip › molecules-1944606-Supplementary .pdf]

Supplementary Material

Supplementary Figure S1. ^{1}H-NMR spectrum of ardisiapunine B **(1)** in pyridine

Supplementary Figure S2. ^{13}C-NMR spectrum of ardisiapunine B **(1)** in pyridine

Supplementary Figure S3. DEPT spectrum of ardisiapunine B **(1)** in pyridine

Supplementary Figure S4. HSQC spectrum of ardisiapunine B **(1)** in pyridine

Supplementary Figure S5. HMBC spectrum of ardisiapunine B **(1)** in pyridine

Supplementary Figure S6. ^{1}H-^{1}H COSY spectrum of ardisiapunine B **(1)** in pyridine

Supplementary Figure S7. NOESY spectrum of ardisiapunine B **(1)** in pyridine

Supplementary Figure S8. HR-ESI-MS spectrum of ardisiapunine B **(1)**

Supplementary Figure S9. HPLC-UV chromatogram of ardisiapunine B **(1)**

Supplementary Figure S10. HPLC-ELSD chromatogram of Ardisiapunine B **(1)**

Supplementary Figure S11. Ultraviolet full wavelength scanning spectrum of ardisiapunine B **(1)**

Supplementary Figure S12. IR spectrum of ardisiapunine B **(1)**

Supplementary Figure S13. ^{1}H-NMR spectrum of ardisiapunine C **(2)** in pyridine

Supplementary Figure S14. ^{13}C-NMR spectrum of ardisiapunine C **(2)** in pyridine

Supplementary Figure S15. DEPT spectrum of ardisiapunine C **(2)** in pyridine

Supplementary Figure S16. HSQC spectrum of ardisiapunine C **(2)** in pyridine

Supplementary Figure S17. HMBC spectrum of ardisiapunine C **(2)** in pyridine

Supplementary Figure S18. ^{1}H-^{1}H COSY spectrum of ardisiapunine C **(2)** in pyridine

Supplementary Figure S19. NOESY spectrum of ardisiapunine C **(2)** in pyridine

Supplementary Figure S20. HR-ESI-MS spectrum of ardisiapunine C **(2)**

Supplementary Figure S21. HPLC-UV chromatogram of ardisiapunine C **(2)**

Supplementary Figure S22. HPLC-ELSD chromatogram of ardisiapunine C **(2)**

Supplementary Figure S23. Ultraviolet full wavelength scanning spectrum of ardisiapunine C **(2)**

Supplementary Figure S24. IR spectrum of ardisiapunine C **(2)**

Supplementary Figure S25. ^{1}H-NMR spectrum of **2-DNP** in pyridine

Supplementary Figure S26. ^{13}C-NMR spectrum of **2-DNP** in pyridine

Supplementary Figure S27. DEPT spectrum of **2-DNP** in pyridine

Supplementary Figure S28. HSQC spectrum of **2-DNP** in pyridine

Supplementary Figure S29. HMBC spectrum of **2-DNP** in pyridine

Supplementary Figure S30. ^{1}H-^{1}H COSY spectrum of **2-DNP** in pyridine

Supplementary Figure S31. NOESY spectrum of **2-DNP** in pyridine

Supplementary Figure S32. HR-ESI-MS spectrum of **2-DNP**

Supplementary Figure S33. HPLC-UV chromatogram of **2-DNP**

Supplementary Figure S34. HPLC-ELSD chromatogram of **2-DNP**

Supplementary Figure S35. Ultraviolet full wavelength scanning spectrum of **2-DNP**

Supplementary Figure S36. IR spectrum of **2-DNP**

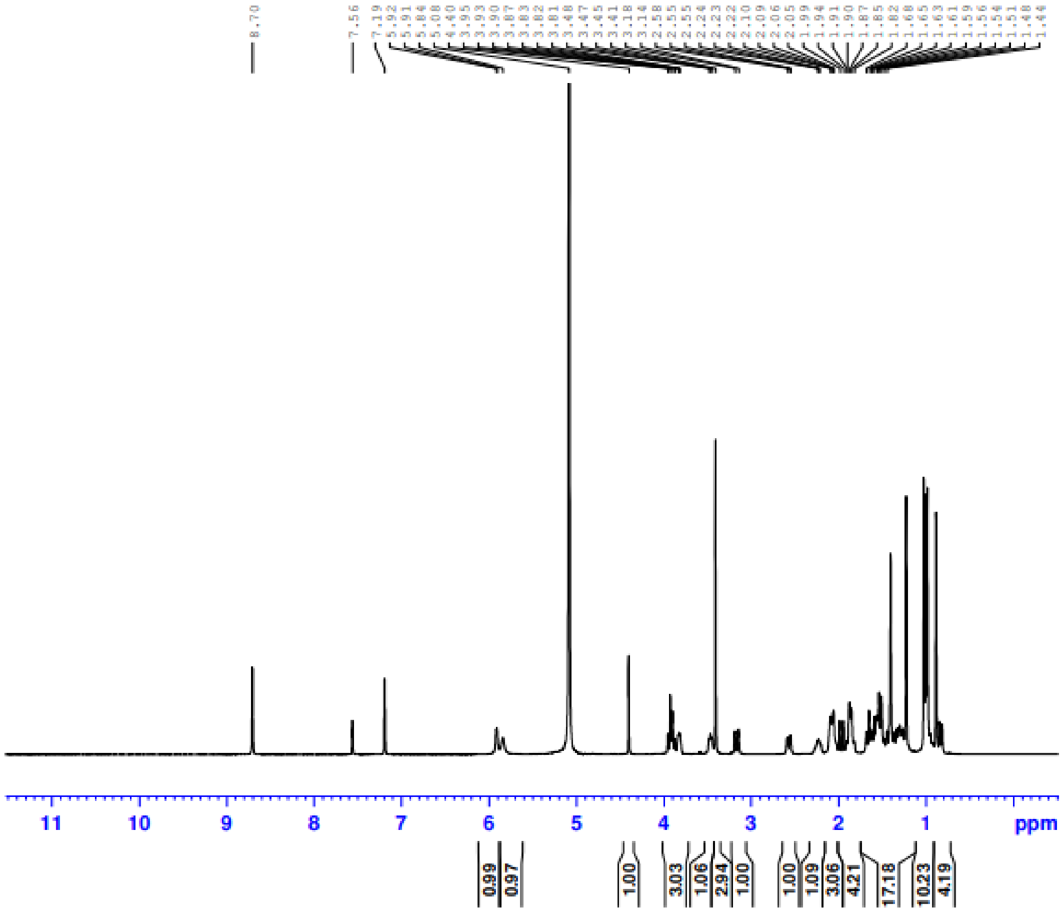

Supplementary Figure S1. ^{1}H-NMR spectrum of ardisiapunine B (**1**) in pyridine.

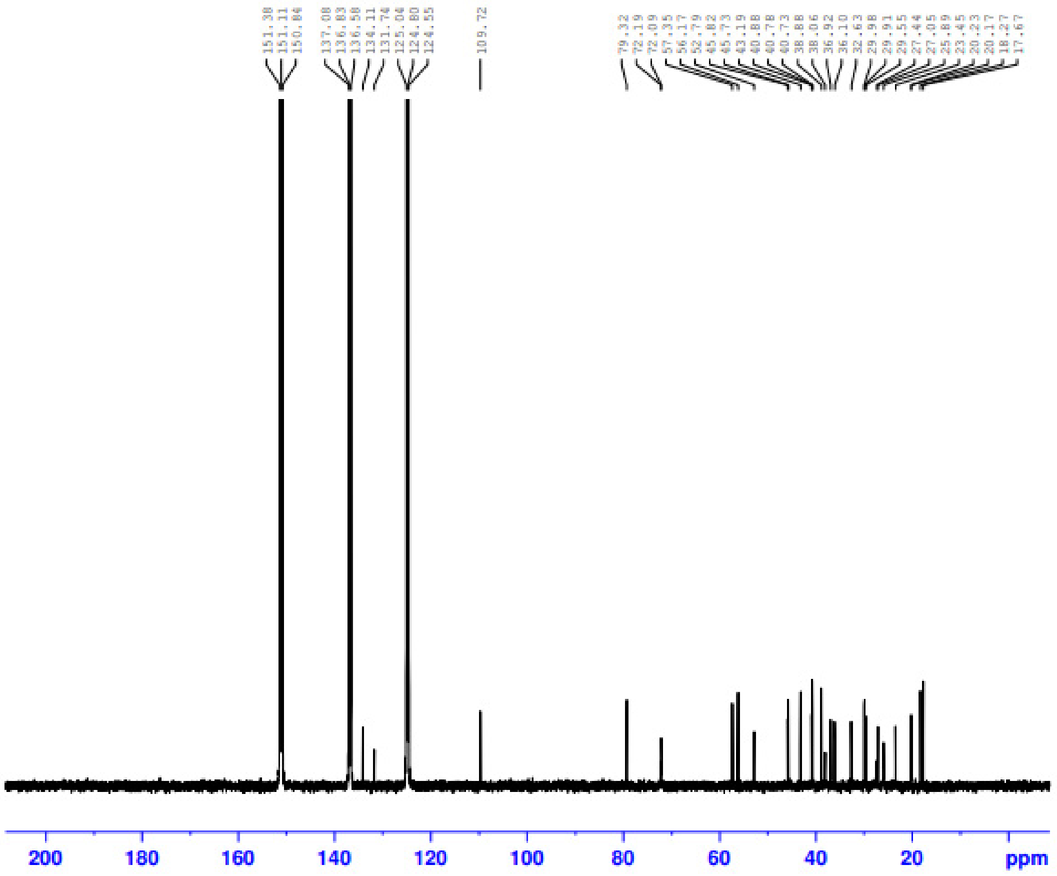

Supplementary Figure S2. ^{13}C-NMR spectrum of ardisiapunine B (**1**) in pyridine.

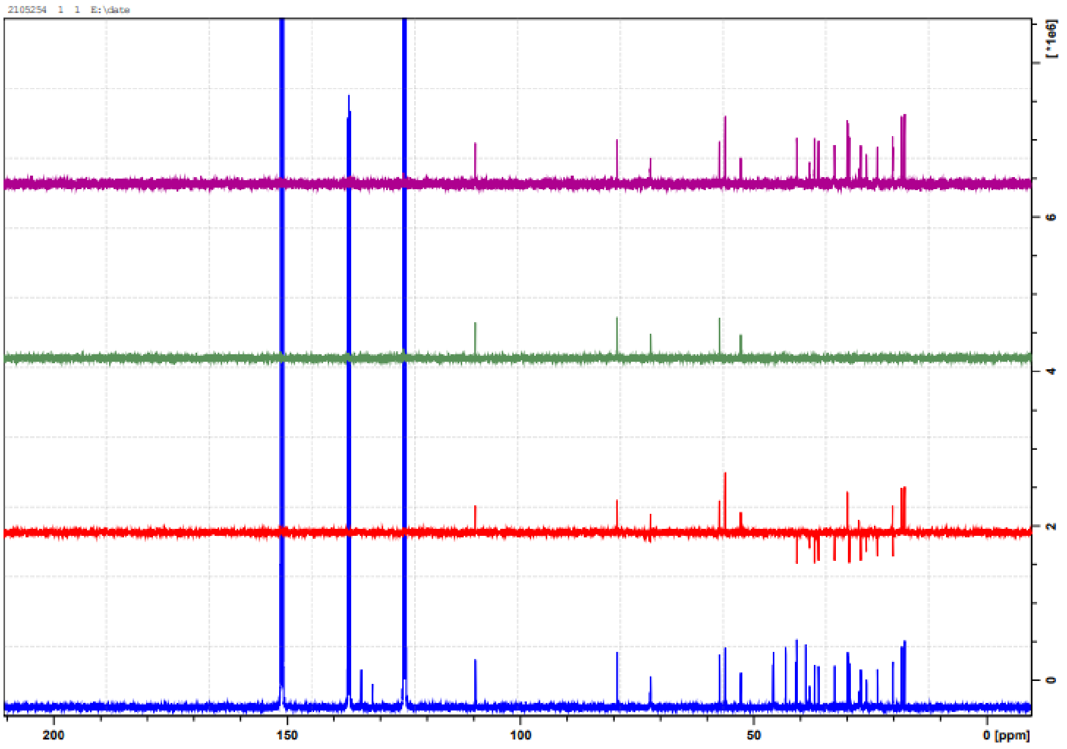

Supplementary Figure S3. DEPT spectrum of ardisiapunine B (**1**) in pyridine.

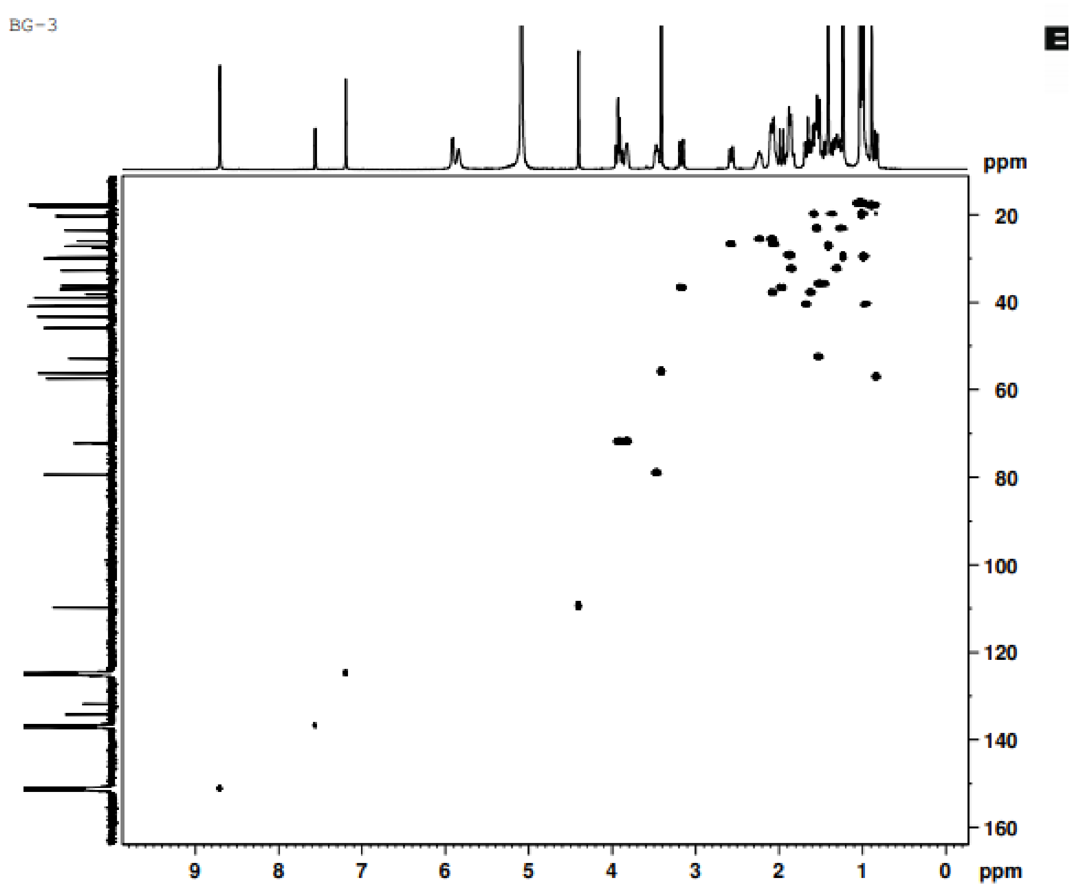

Supplementary Figure S4. HSQC spectrum of ardisiapunine B (**1**) in pyridine

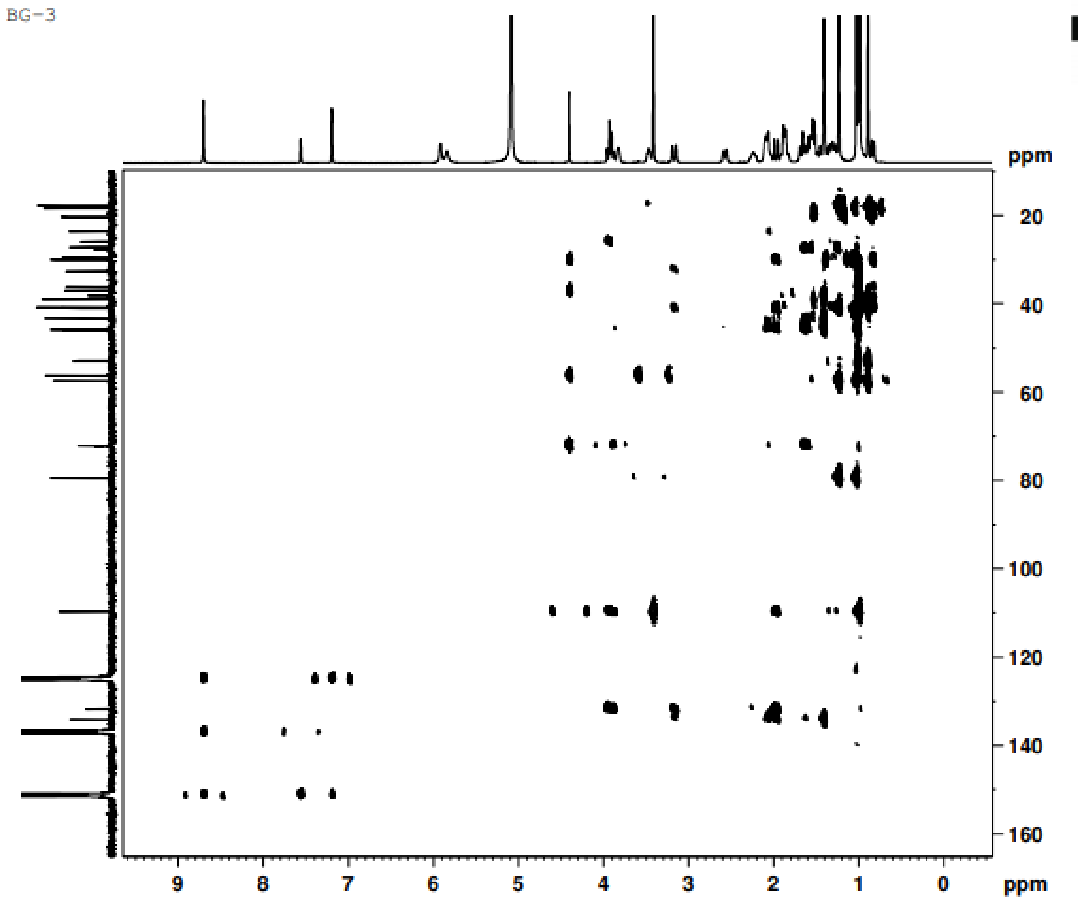

Supplementary Figure S5. HMBC spectrum of ardisiapunine B (**1**) in pyridine.

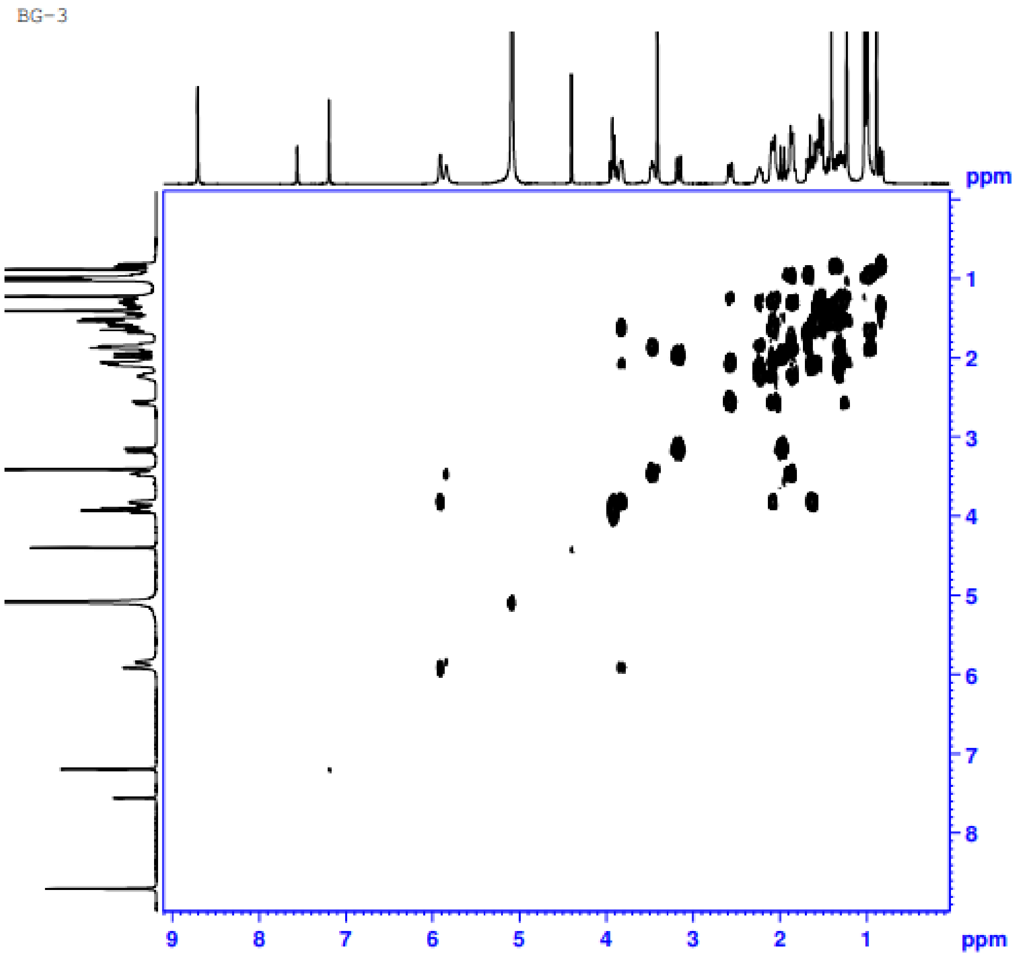

Supplementary Figure S6. ^{1}H-^{1}H COSY spectrum of ardisiapunine B (**1**) in pyridine.

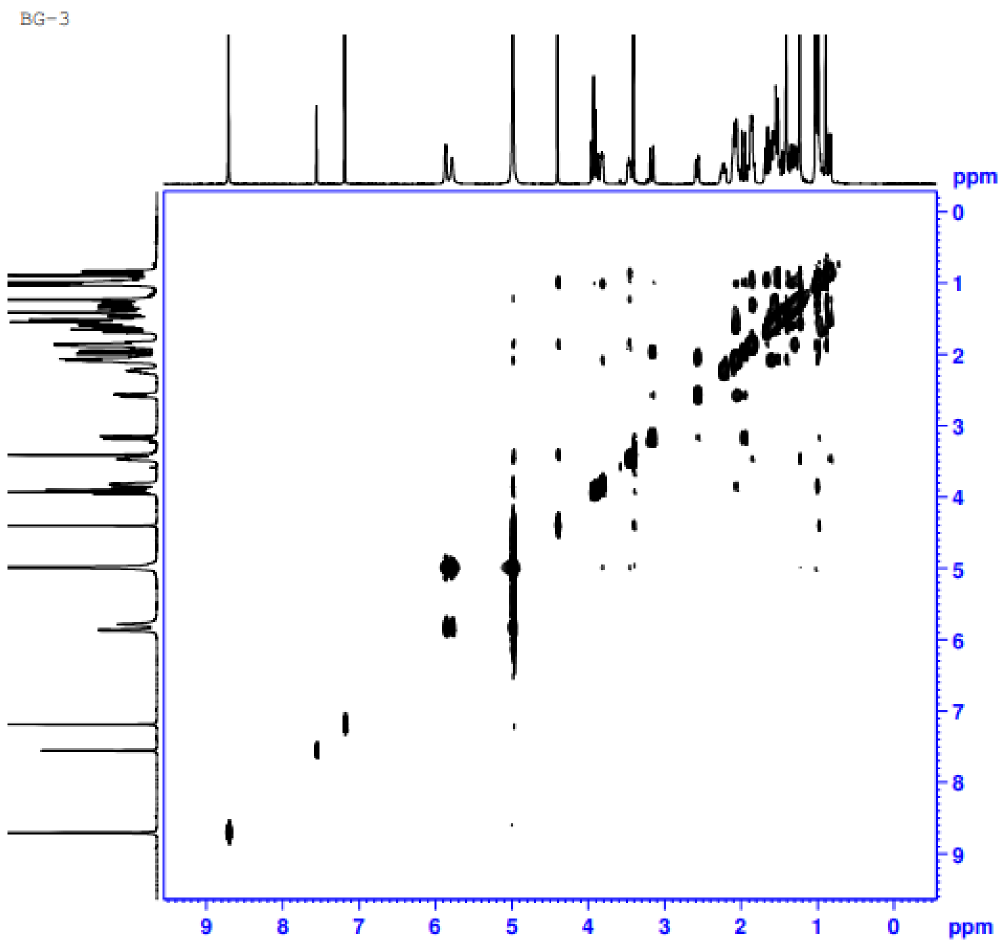

Supplementary Figure S7. NOESY spectrum of ardisiapunine B (**1**) in pyridine.

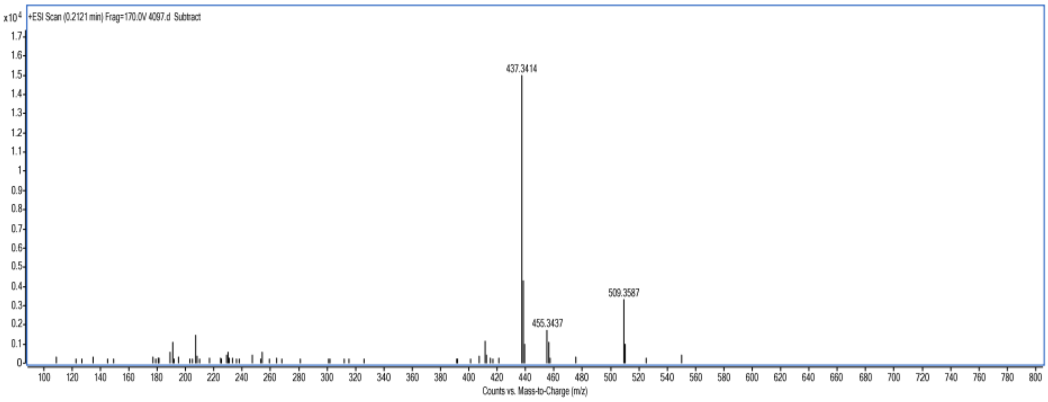

Supplementary Figure S8. HR-ESI-MS spectrum of ardisiapunine B (**1**).

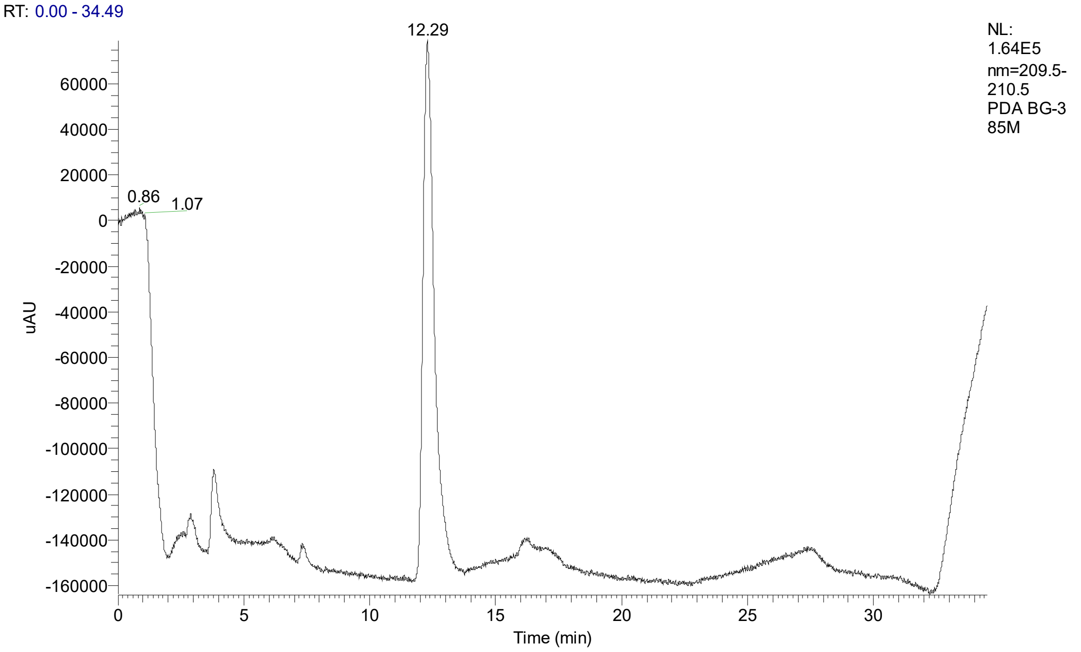

Supplementary Figure S9. HPLC-UV chromatogram of ardisiapunine B (**1**).

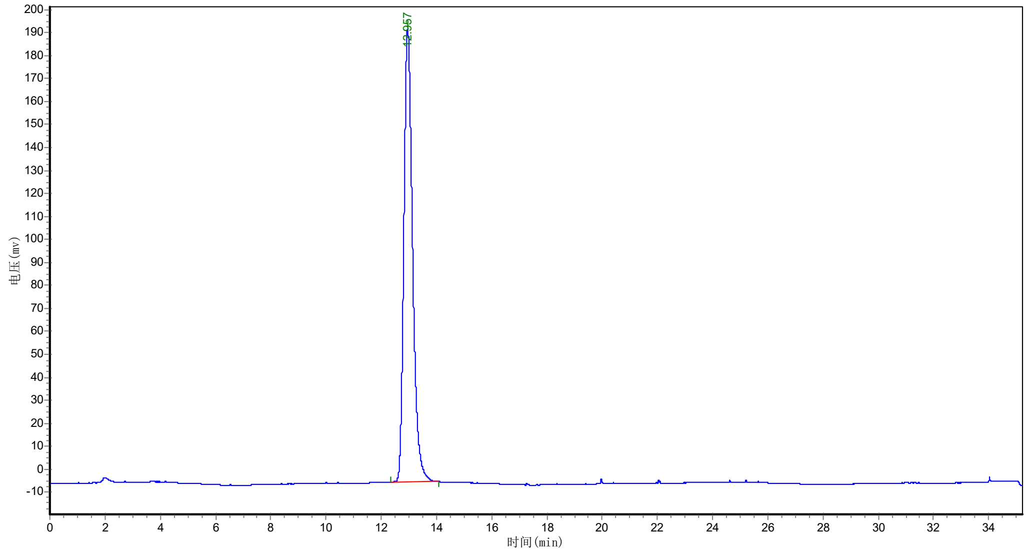

Supplementary Figure S10. HPLC-ELSD chromatogram of ardisiapunine B (**1**).

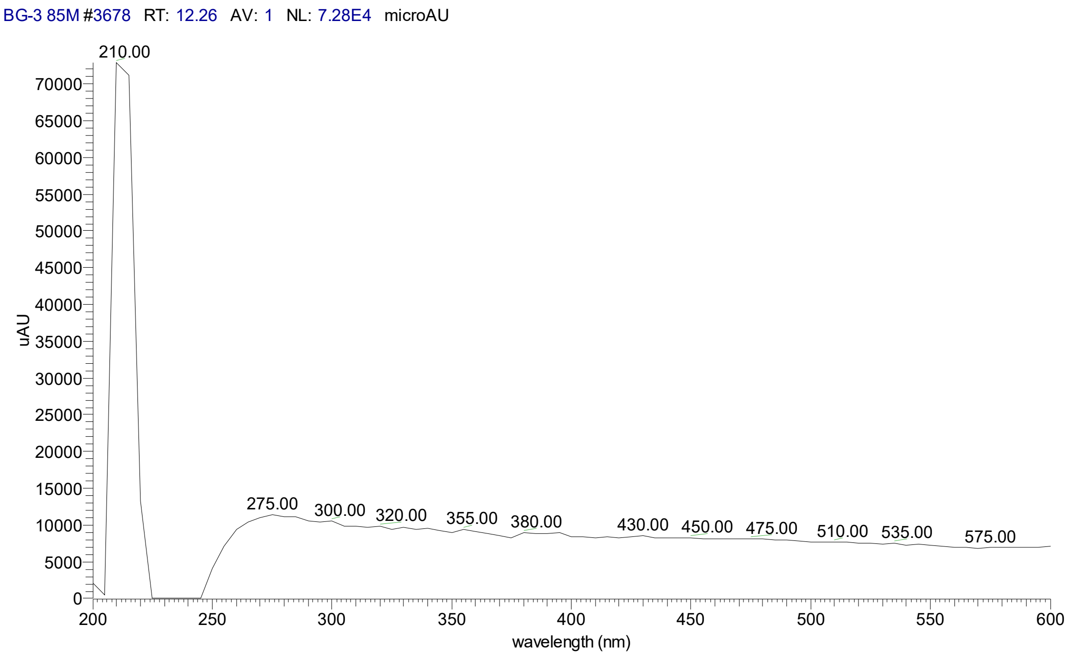

Supplementary Figure S11. Ultraviolet full wavelength scanning spectrum of ardisiapunine B (**1**).

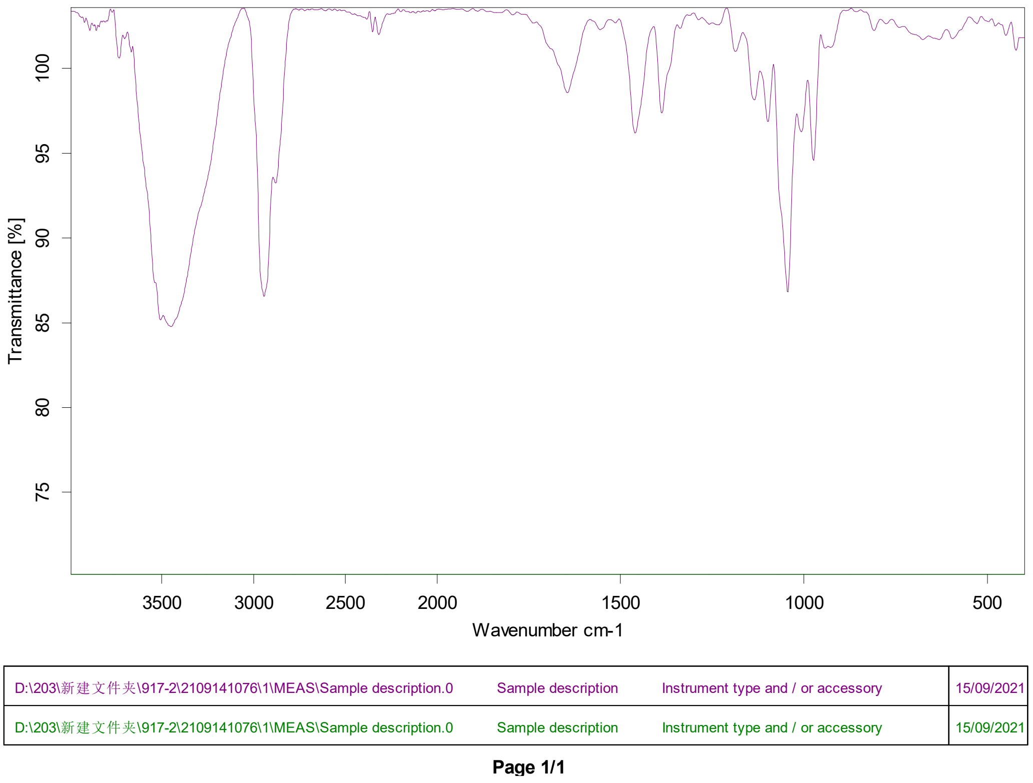

Supplementary Figure S12. IR spectrum of ardisiapunine B (**1**).

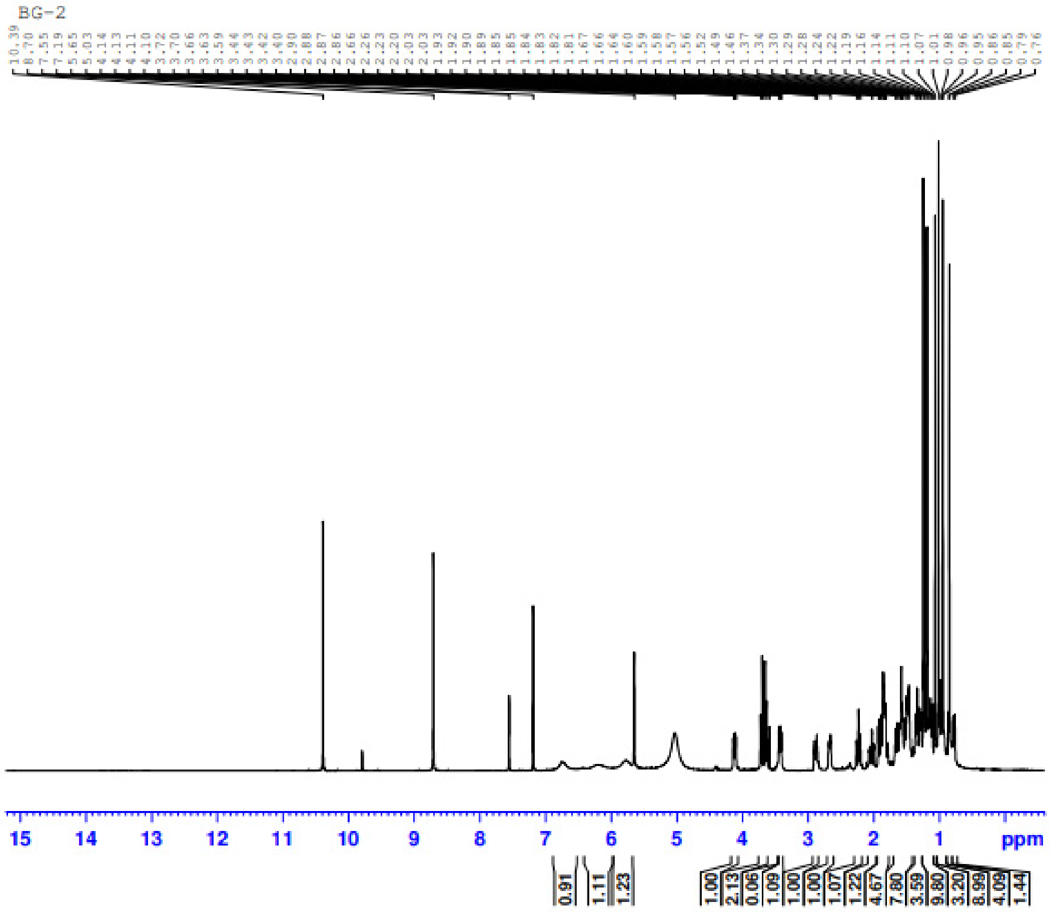

Supplementary Figure S13. ^{1}H-NMR spectrum of ardisiapunine C (**2**) in pyridine.

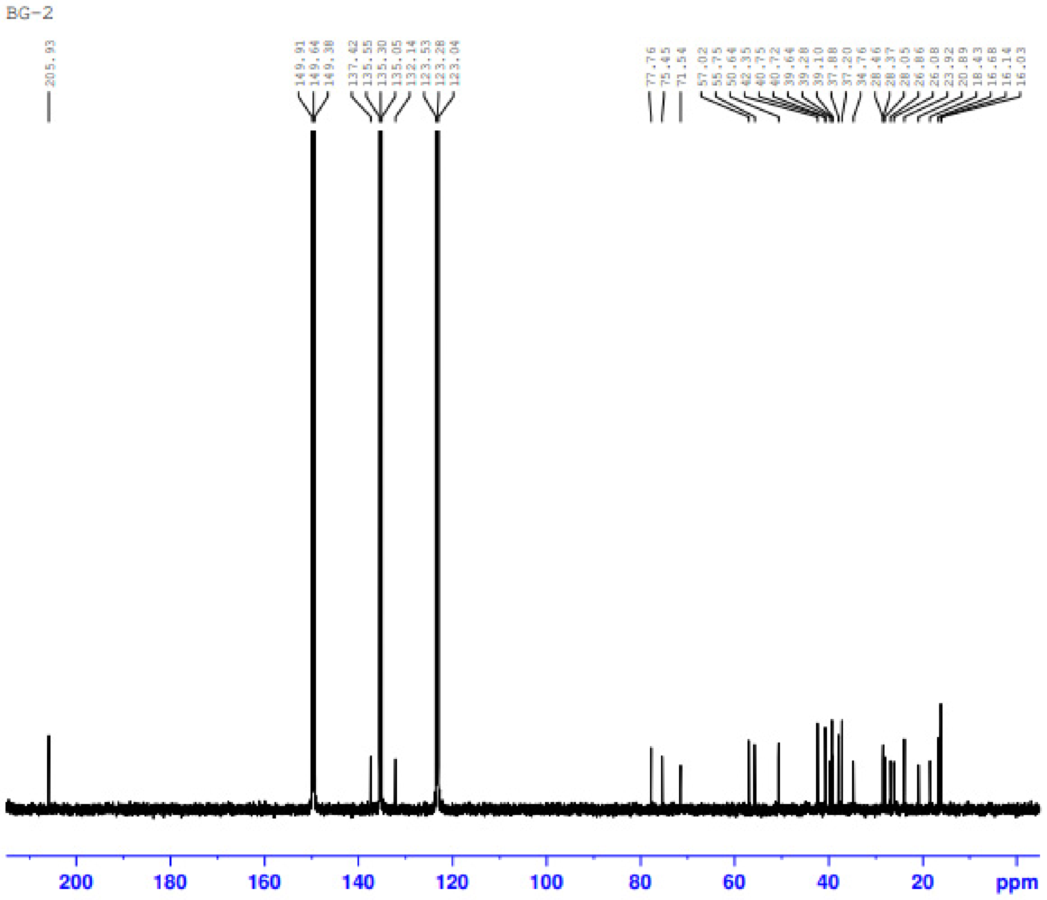

Supplementary Figure S14. ^{13}C-NMR spectrum of ardisiapunine C (**2**) in pyridine.

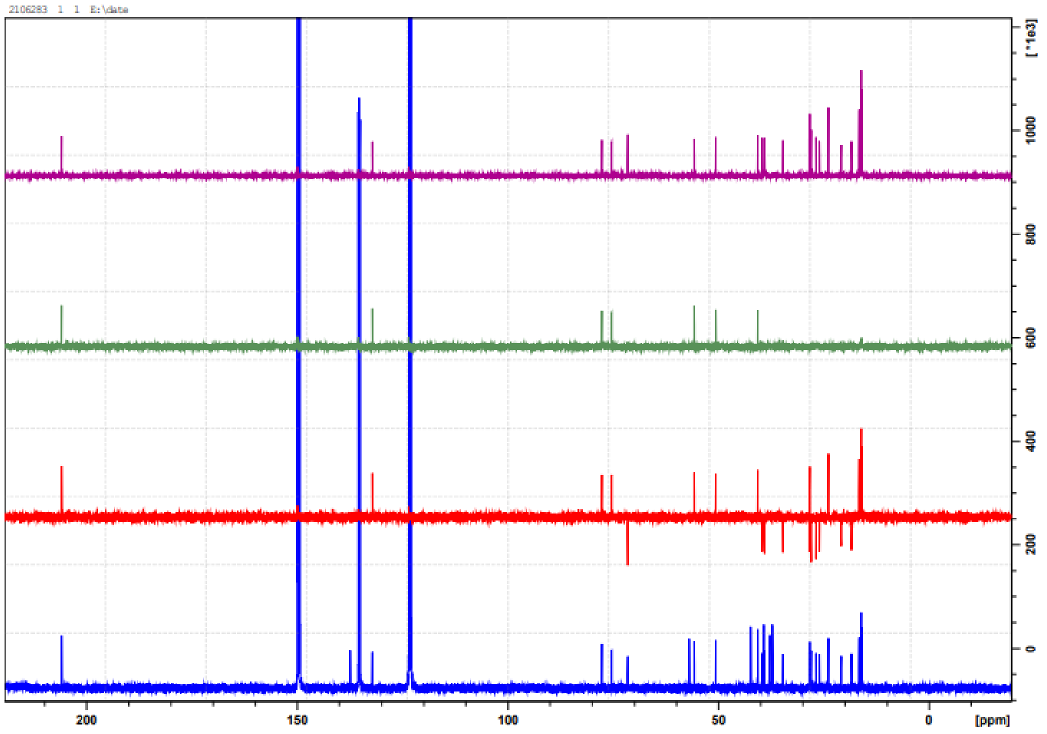

Supplementary Figure S15. DEPT spectrum of ardisiapunine C (**2**) in pyridine.

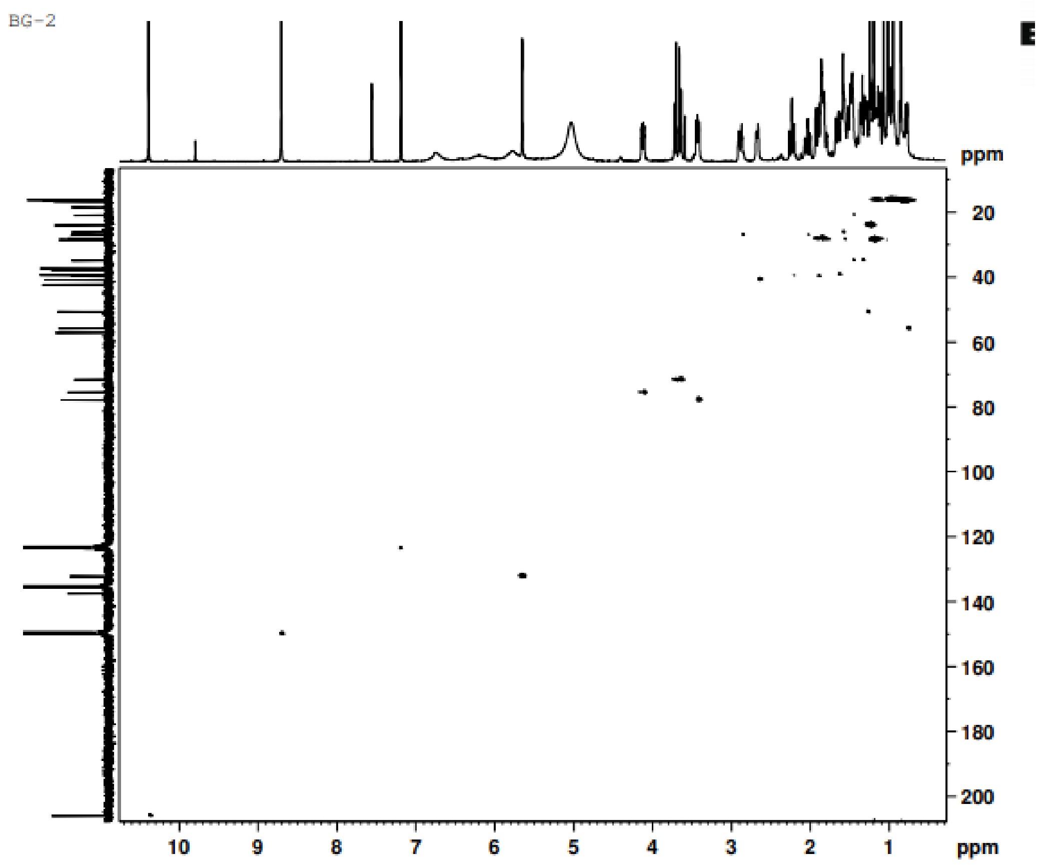

Supplementary Figure S16. HSQC spectrum of ardisiapunine C (**2**) in pyridine.

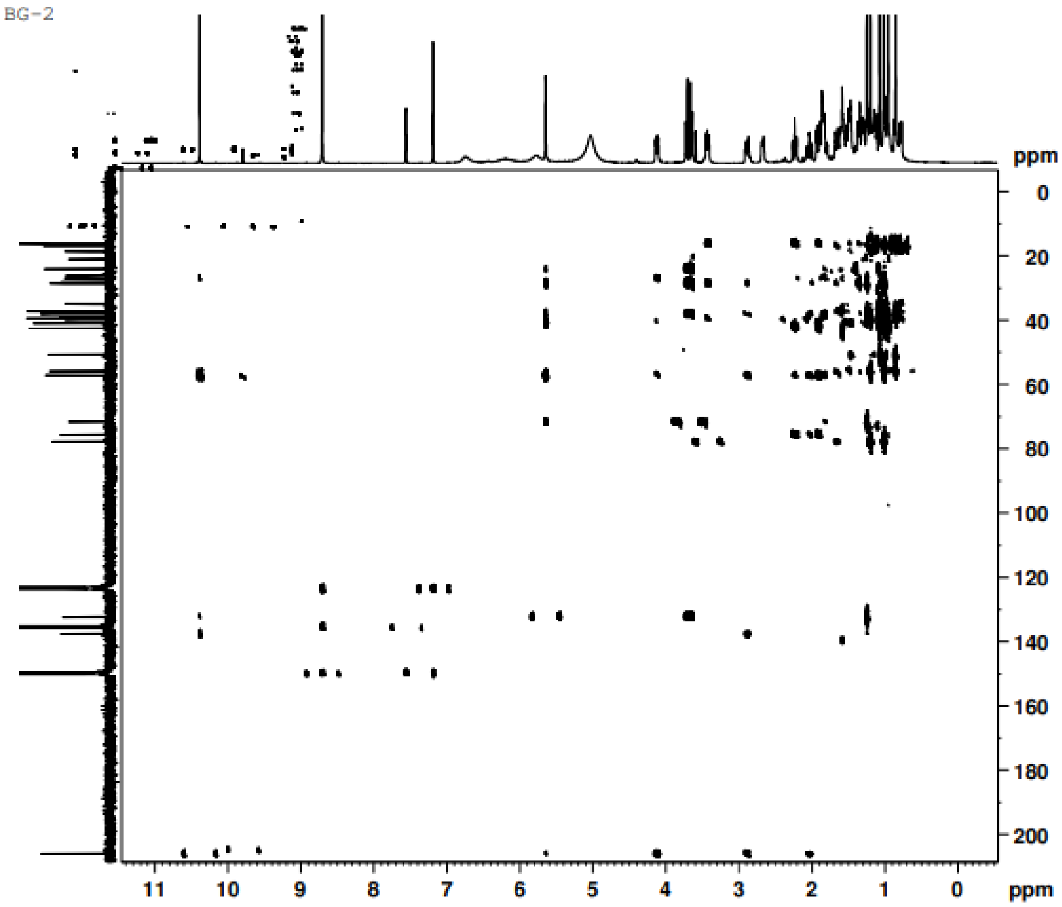

Supplementary Figure S17. HMBC spectrum of ardisiapunine C (**2**) in pyridine.

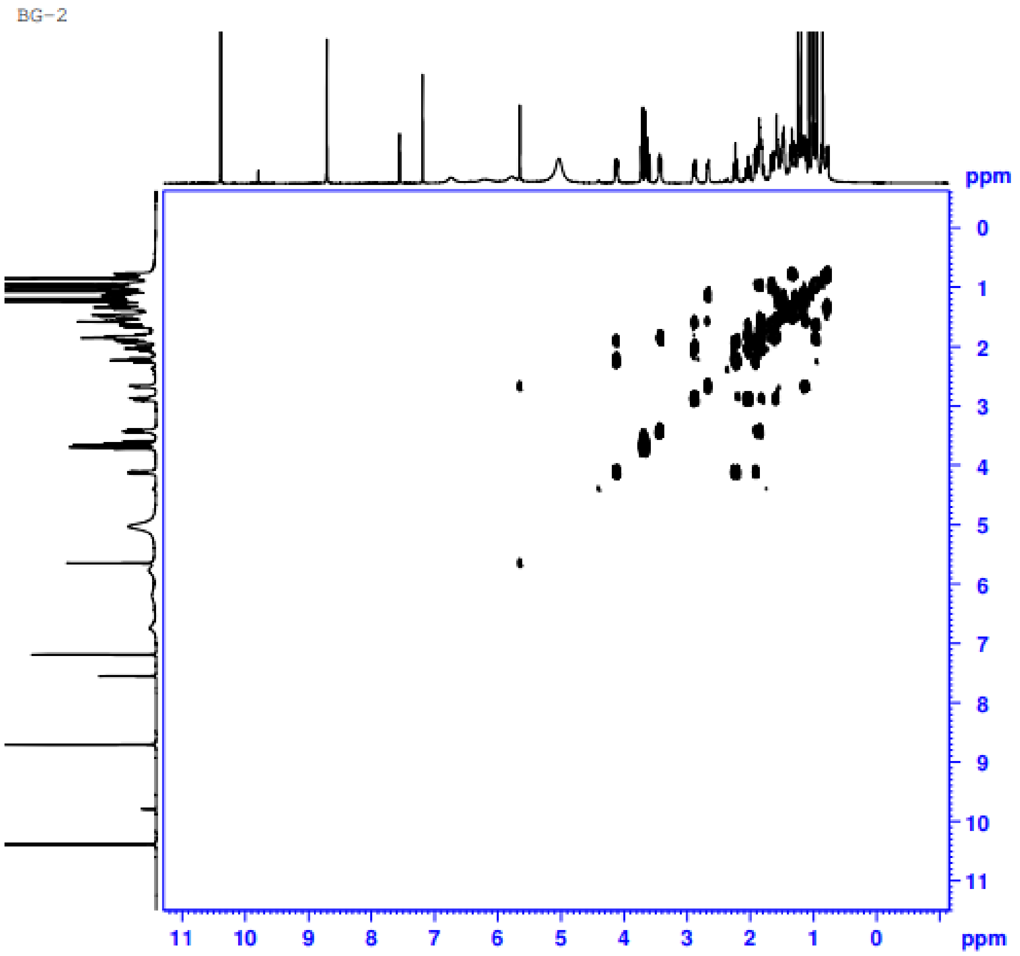

Supplementary Figure S18. ^{1}H-^{1}H COSY spectrum of ardisiapunine C (**2**) in pyridine.

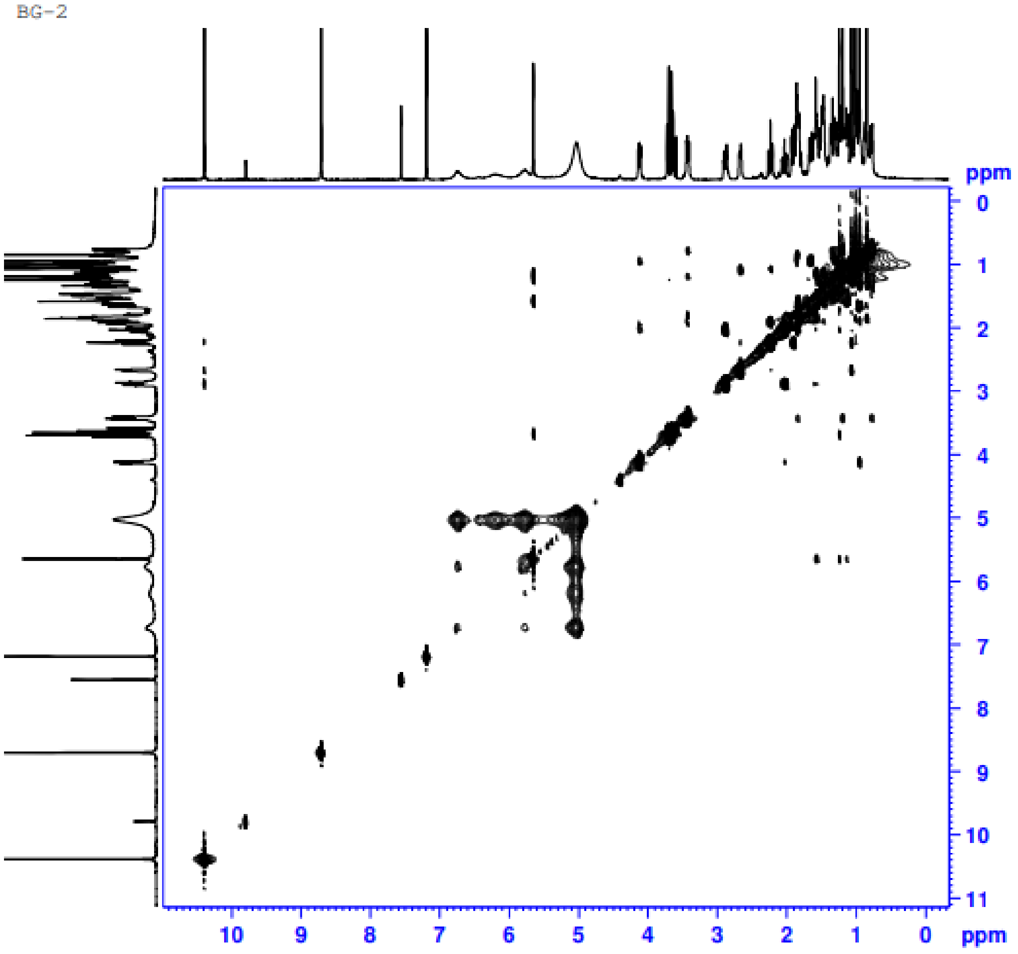

Supplementary Figure S19. NOESY spectrum of ardisiapunine C (**2**) in pyridine.

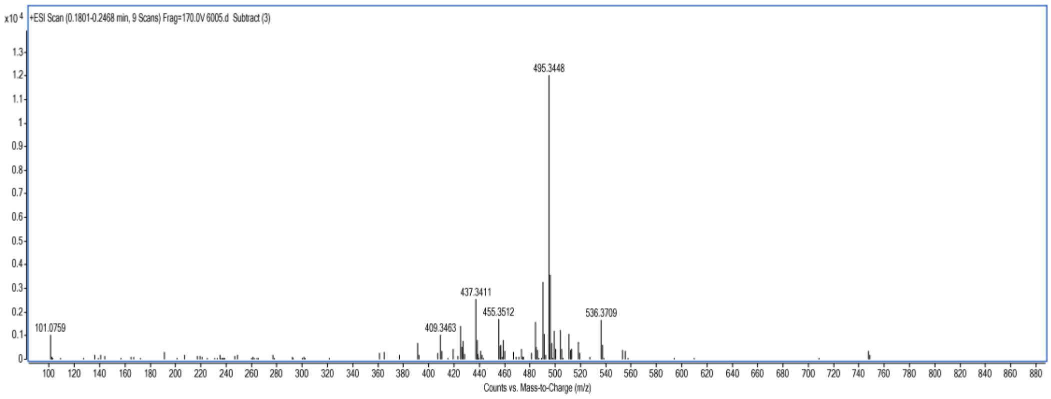

Supplementary Figure S20. HR-ESI-MS spectrum of ardisiapunine C (**2**).

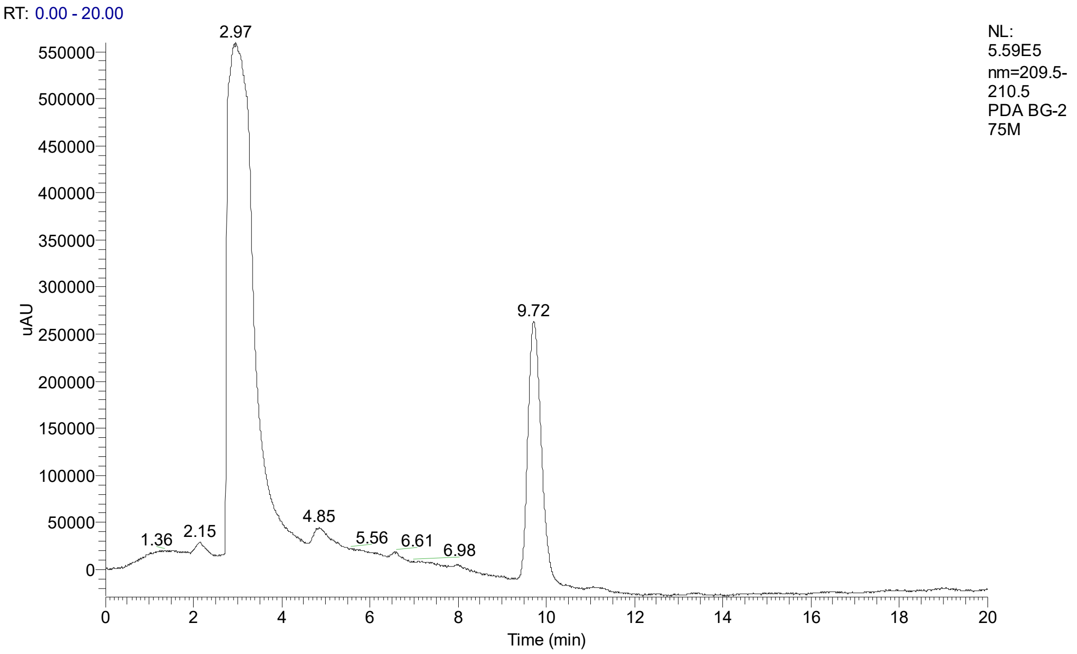

Supplementary Figure S21. HPLC-UV chromatogram of ardisiapunine C (**2**) in DMSO.

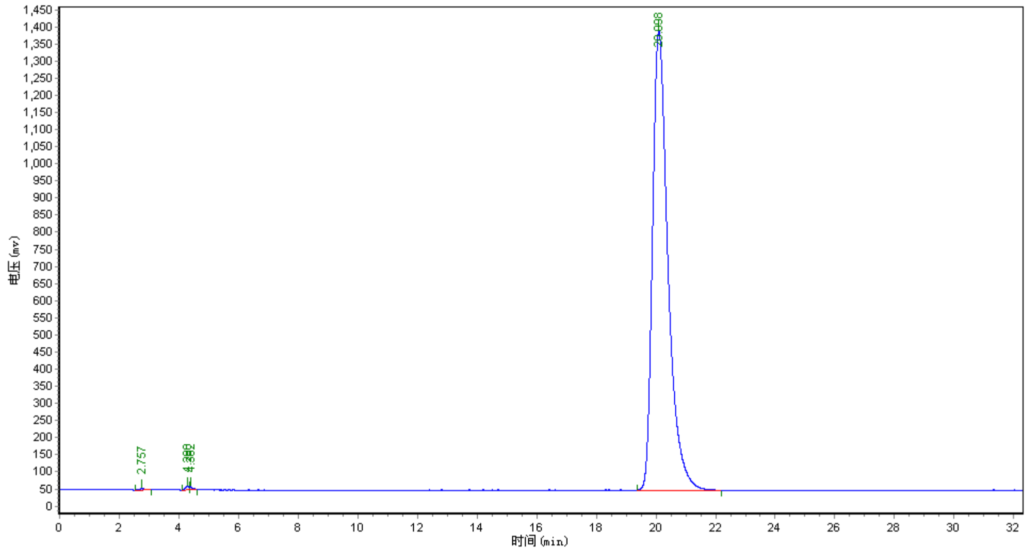

Supplementary Figure S22. HPLC-ELSD chromatogram of Ardisiapunine C (**2**) in DMSO.

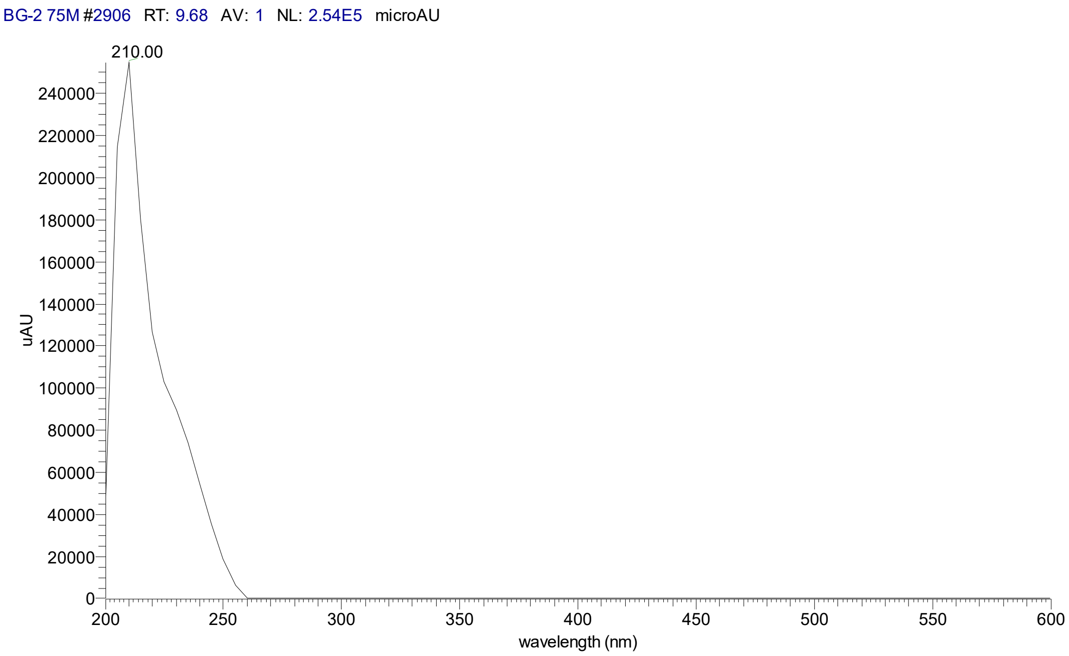

Supplementary Figure S23. Ultraviolet full wavelength scanning spectrum of ardisiapunine C (**2**).

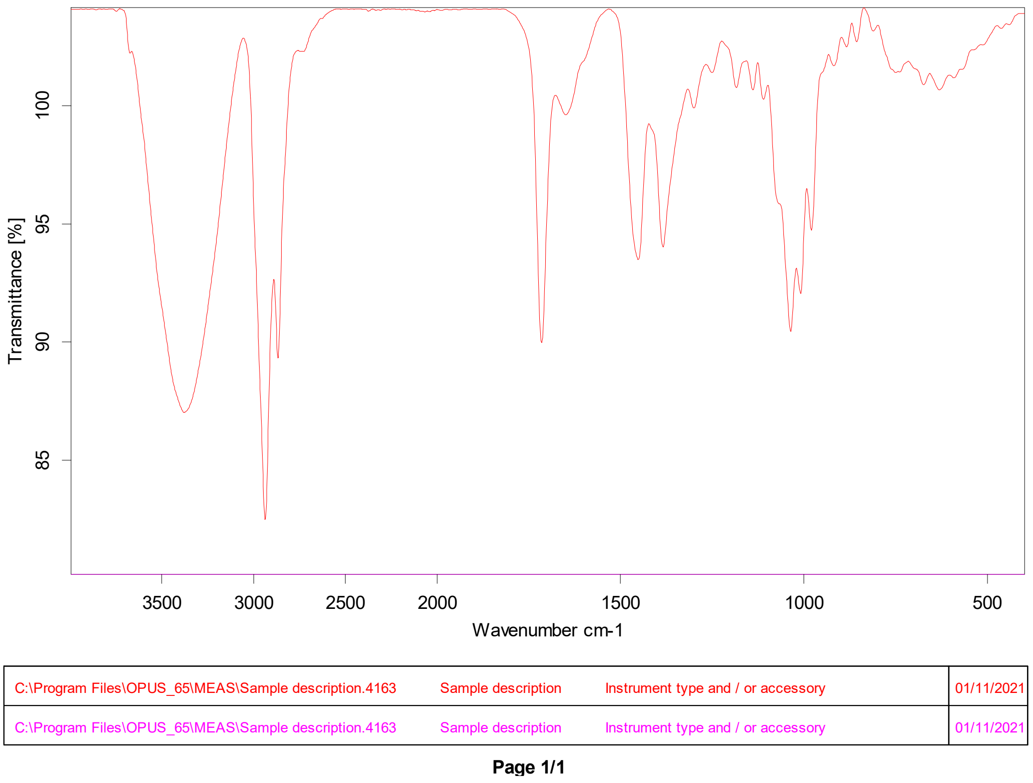

Supplementary Figure S24. IR spectrum of ardisiapunine C (**2**).

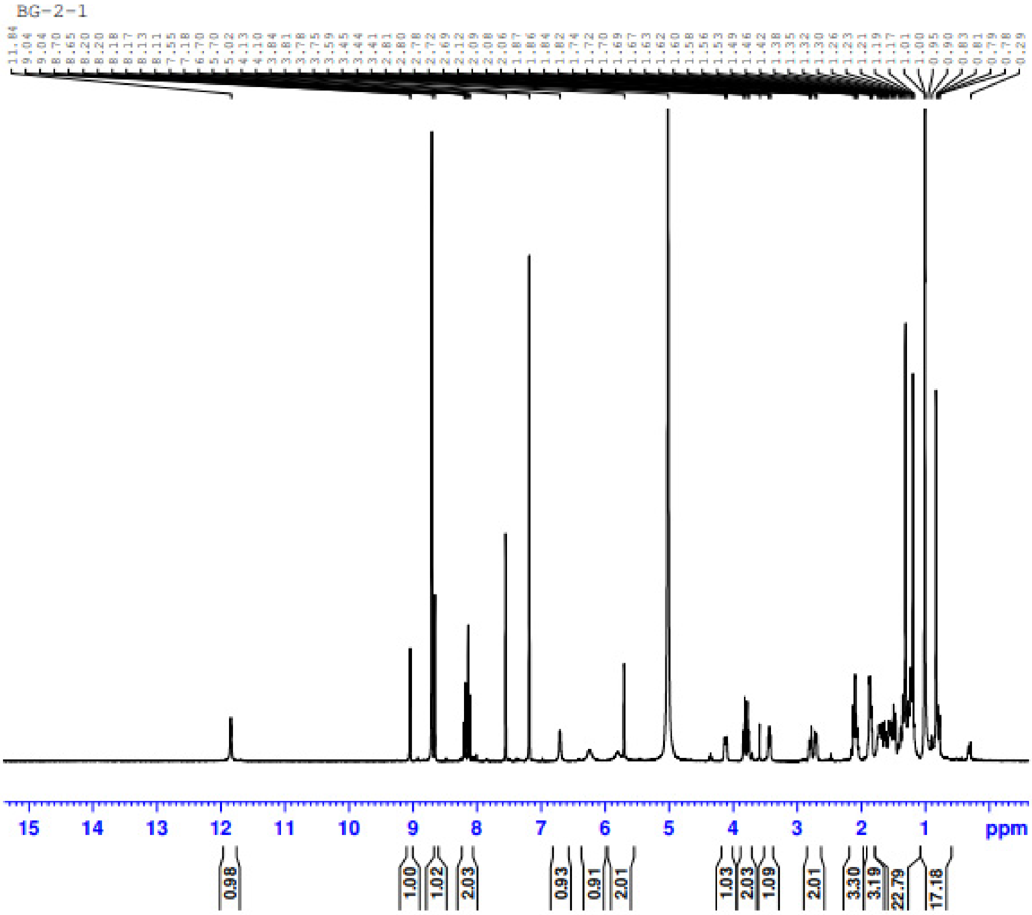

Supplementary Figure S25. ^{1}H-NMR spectrum of **2-DNP** in pyridine.

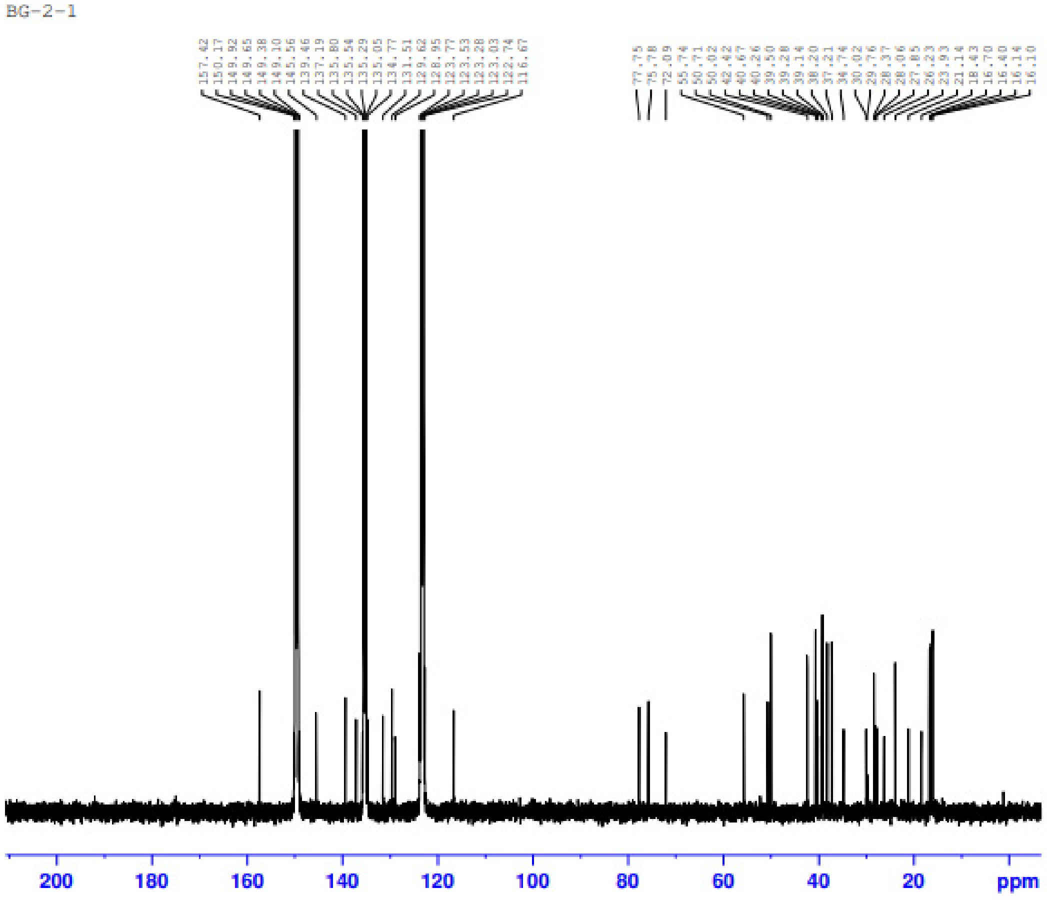

Supplementary Figure S26. ^{13}C-NMR spectrum of **2-DNP** in pyridine.

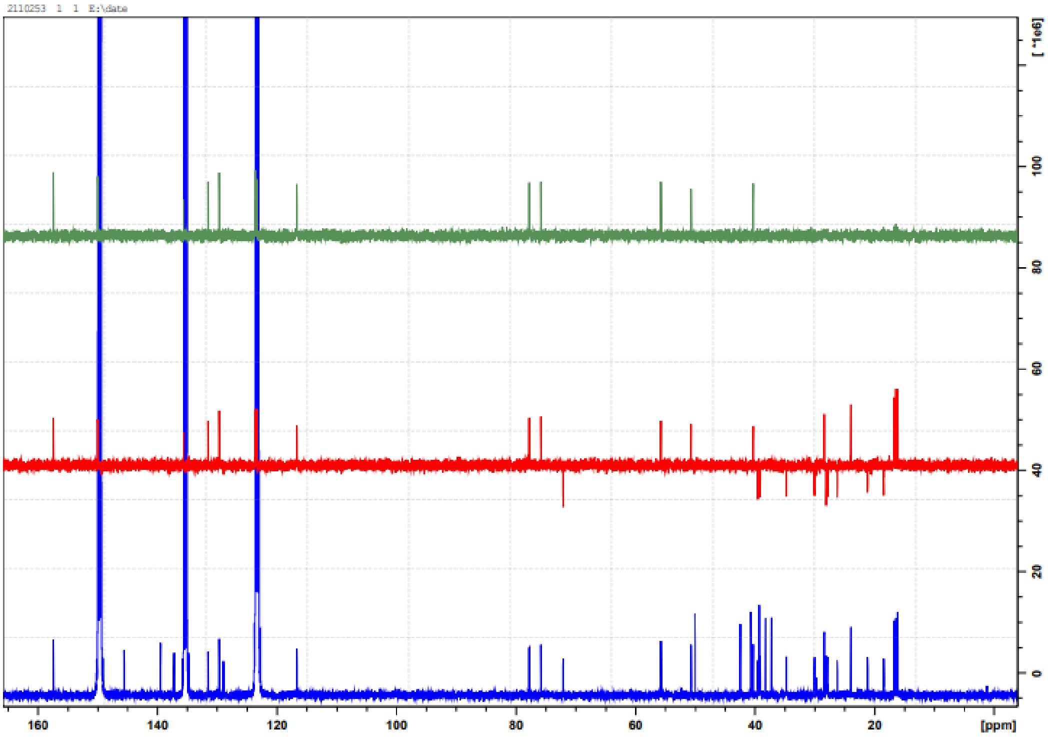

Supplementary Figure S27. DEPT spectrum of **2-DNP** in pyridine.

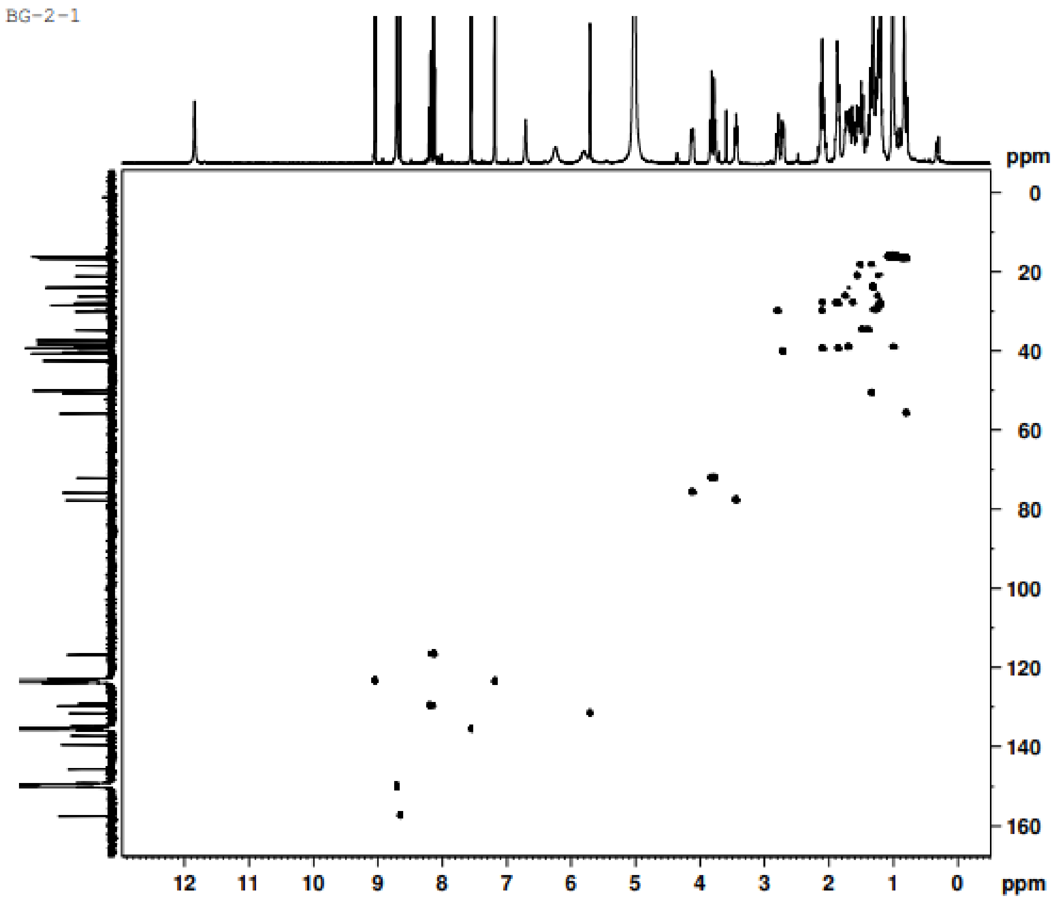

Supplementary Figure S28. HSQC spectrum of **2-DNP** in pyridine.

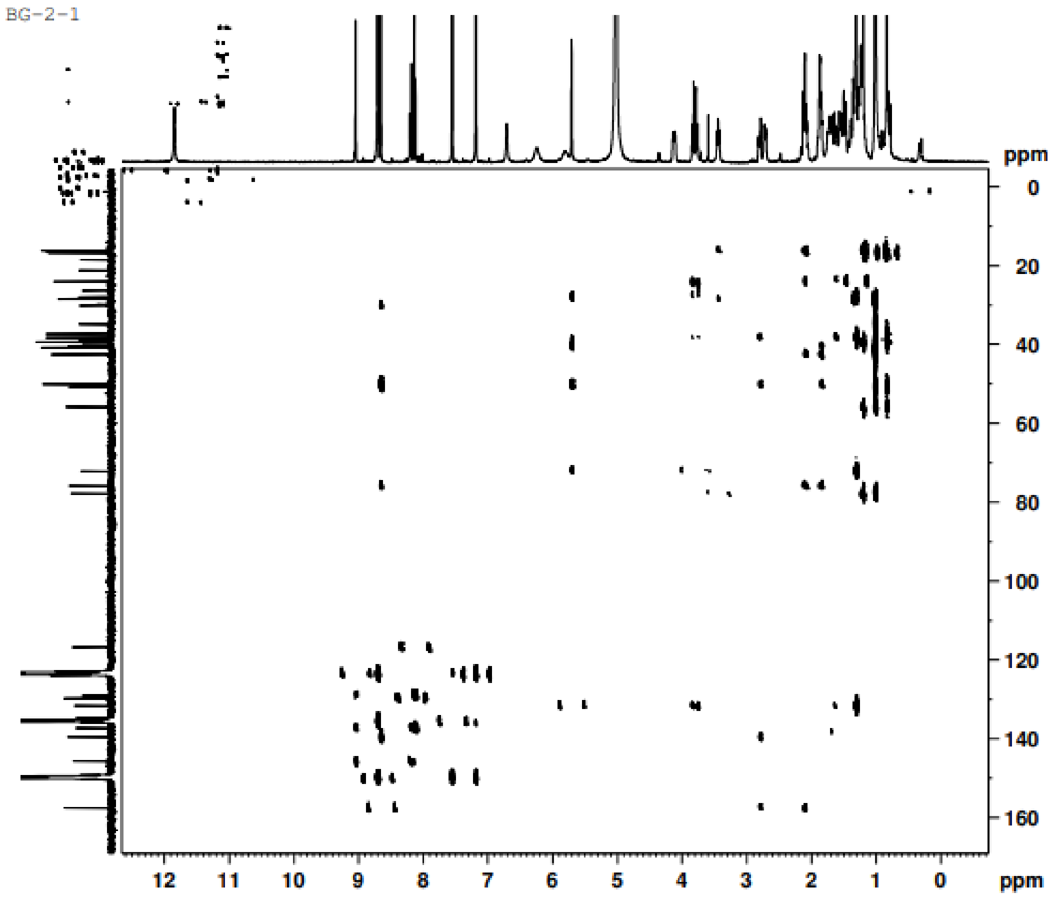

Supplementary Figure S29. HMBC spectrum of **2-DNP** in pyridine.

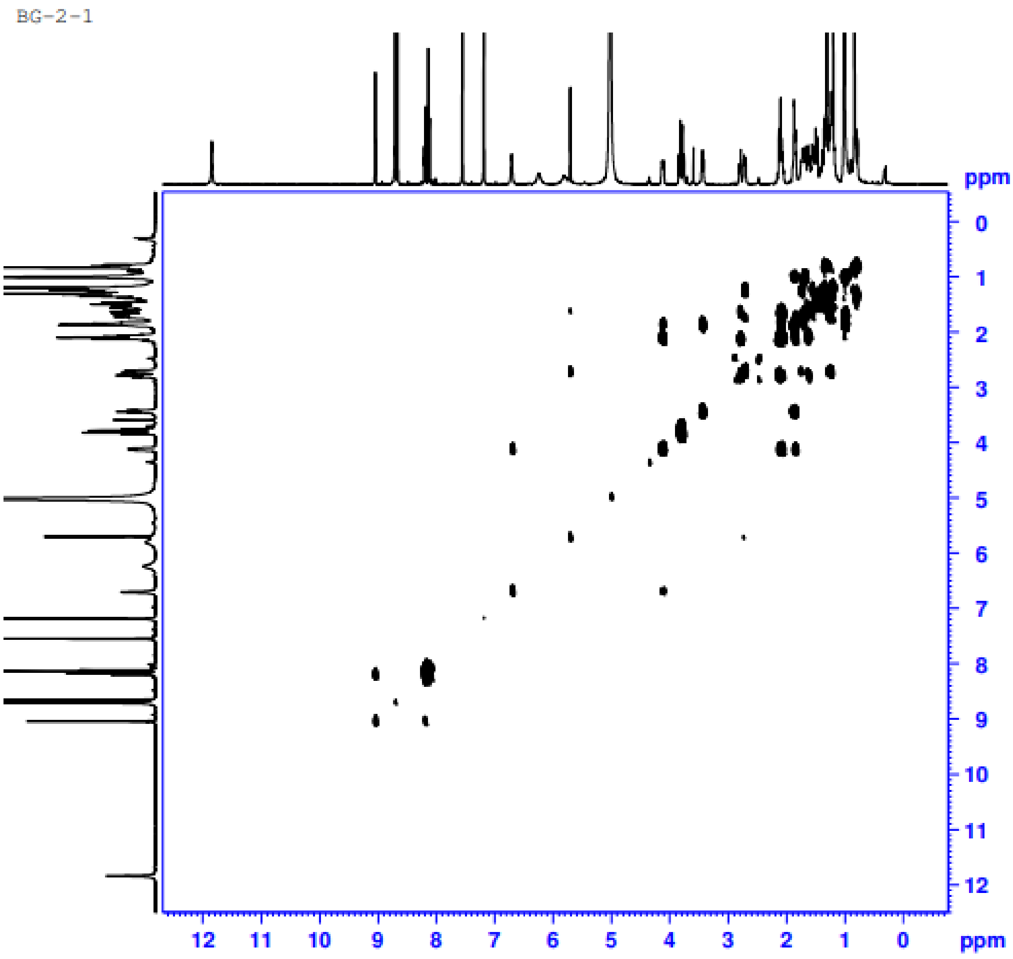

Supplementary Figure S30. ^{1}H-^{1}H COSY spectrum of **2-DNP** in pyridine.

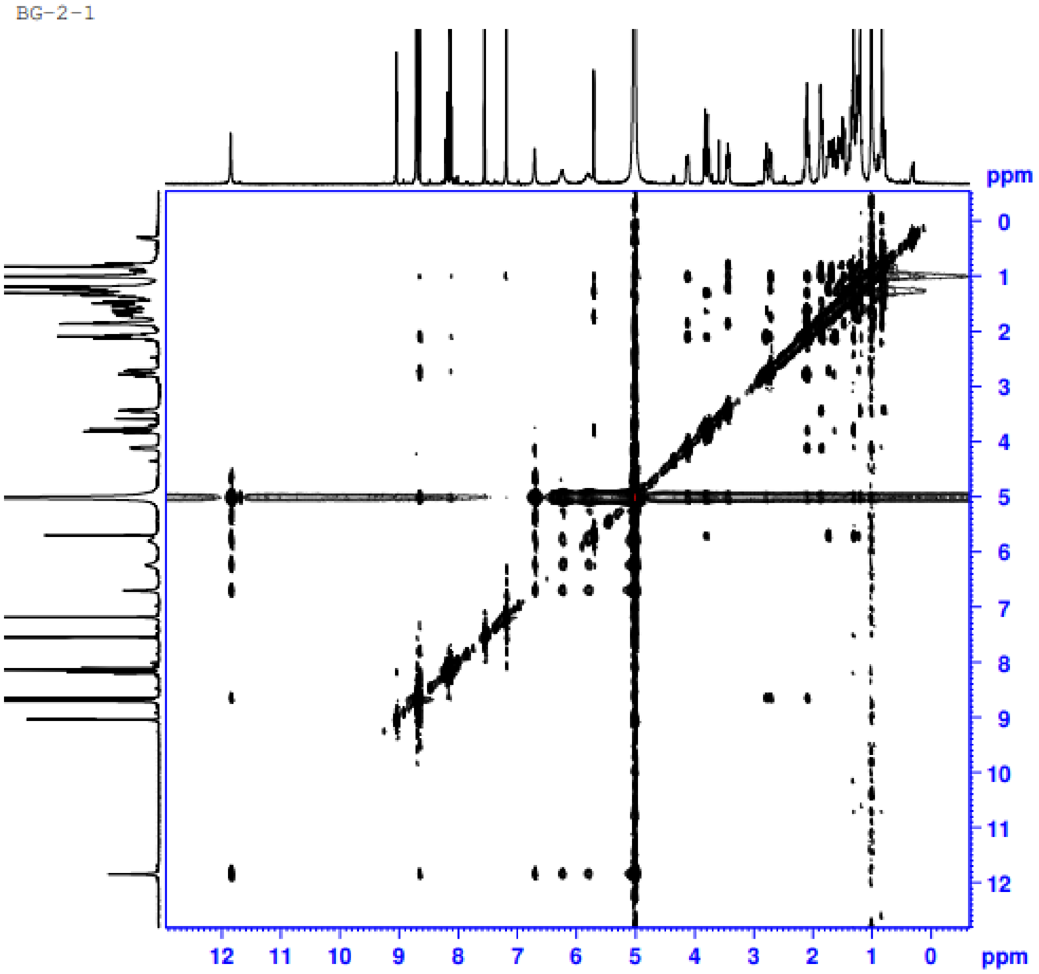

Supplementary Figure S31. NOESY spectrum of **2-DNP** in pyridine.

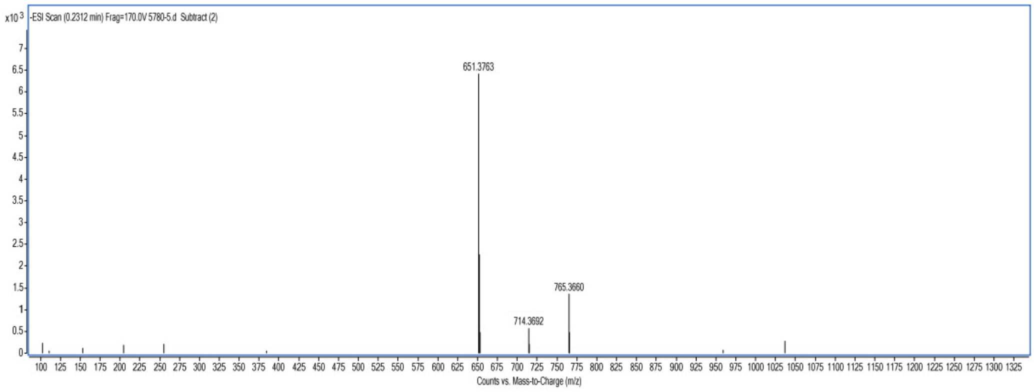

Supplementary Figure S32. HR-ESI-MS spectrum of **2-DNP**.

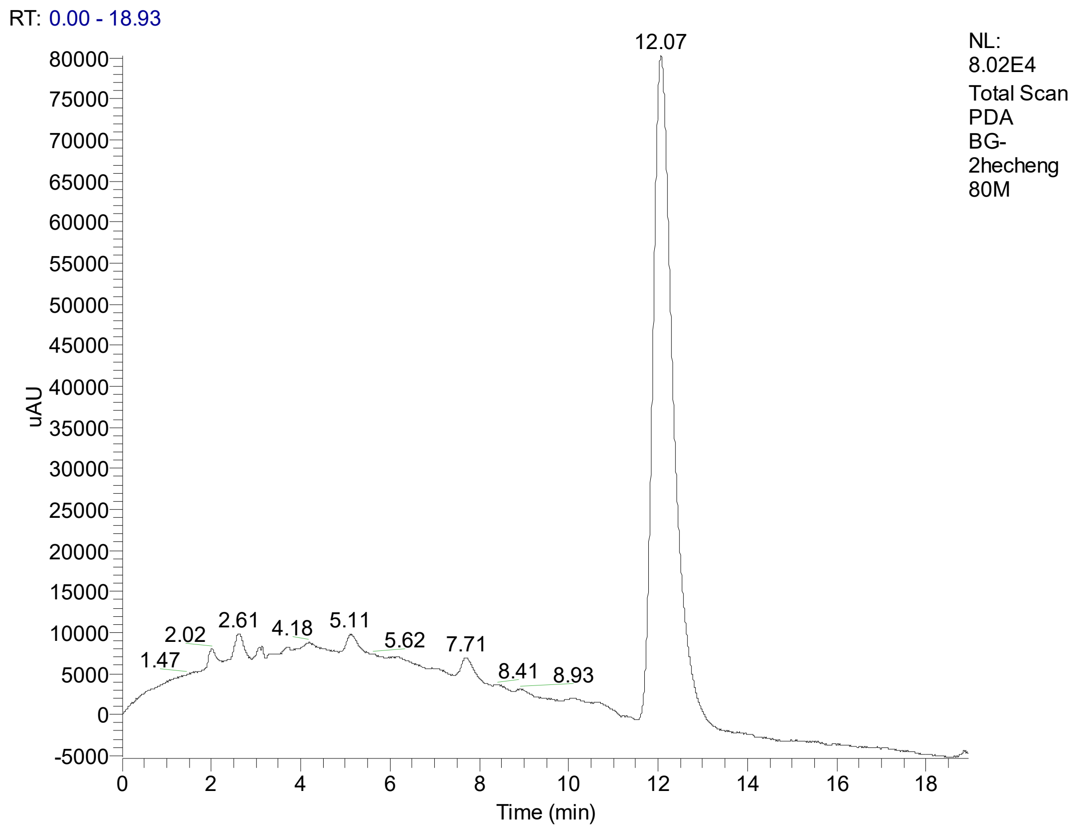

Supplementary Figure S33. HPLC-UV chromatogram of **2-DNP**.

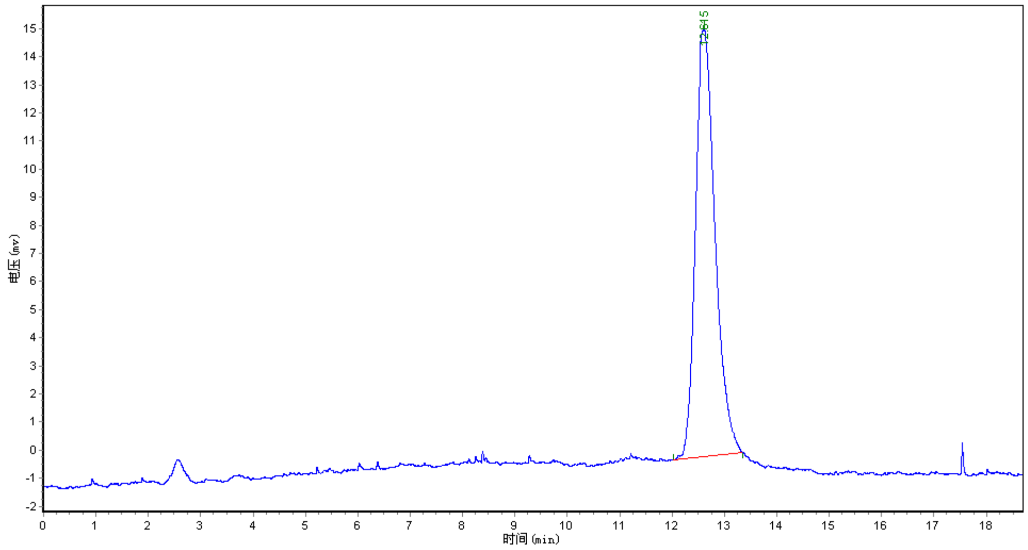

Supplementary Figure S34. HPLC-ELSD chromatogram of **2-DNP**.

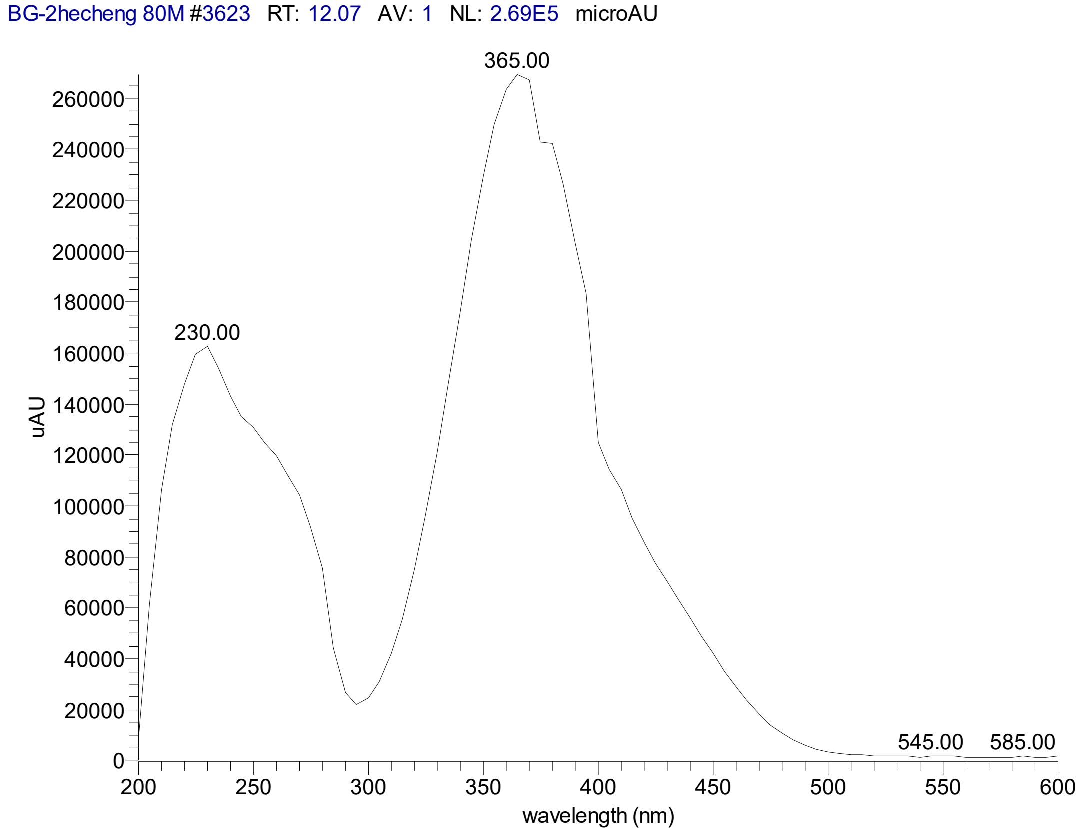

Supplementary Figure S35. Ultraviolet full wavelength scanning spectrum of **2-DNP**.

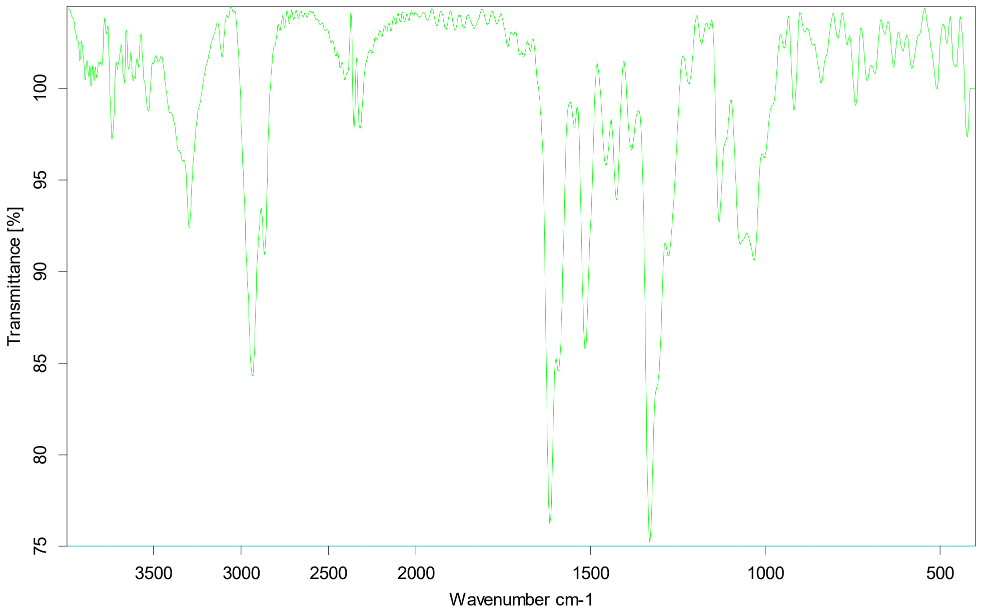

C:\Program Files\OPUS_65\MEAS\Sample description.4164	Sample description	Instrument type and / or accessory	01/11/2021
C:\Program Files\OPUS_65\MEAS\Sample description.4164	Sample description	Instrument type and / or accessory	01/11/2021

Page 1/1

Supplementary Figure S36. IR spectrum of **2-DNP**.
